# Is sirolimus a therapeutic option for patients with progressive pulmonary lymphangioleiomyomatosis?

**DOI:** 10.1186/1465-9921-12-66

**Published:** 2011-05-21

**Authors:** Claus Neurohr, Anna L Hoffmann, Patrick Huppmann, Vivian A Herrera, Franziska Ihle, Stefan Leuschner, Werner von Wulffen, Tobias Meis, Carlos Baezner, Hanno Leuchte, Rainer Baumgartner, Gregor Zimmermann, Juergen Behr

**Affiliations:** 1 Department of Int. Medicine I, Division of Pulmonary Diseases, Klinikum Grosshadern, Ludwig-Maximilians University, Comprehensive Pneumology Center, Marchioninistrasse 15, 81377 Munich, Germany

## Abstract

**Background:**

Lymphangioleiomyomatosis (LAM) is a rare lung disease characterised by progressive airflow obstruction. No effective medical treatment is available but therapy with sirolimus has shown some promise. The aim of this observational study was to evaluate sirolimus in progressive LAM.

**Methods:**

Sirolimus (trough level 5 - 10 ng/ml) was administered to ten female patients (42.4 ± 11.9 years) with documented progression. Serial pulmonary function tests and six-minute-walk-distance (6-MWD) assessments were performed.

**Results:**

The mean loss of FEV_1 _was -2.30 ± 0.52 ml/day before therapy and a significant mean gain of FEV_1 _of 1.19 ± 0.26 ml/day was detected during treatment (p = 0.001). Mean FEV_1 _and FVC at baseline were 1.12 ± 0.15 l (36.1 ± 4.5%pred.) and 2.47 ± 0.25 l (69.2 ± 6.5%pred.), respectively. At three and six months during follow-up a significant increase of FEV_1 _and FVC was demonstrated (3 months ΔFEV_1_: 220 ± 82 ml, p = 0.024; 6 months ΔFEV_1_: 345 ± 58 ml, p = 0.001); (3 months ΔFVC: 360 ± 141 ml, p = 0.031; 6 months ΔFVC: 488 ± 138 ml, p = 0.006). Sirolimus was discontinued in 3 patients because of serious recurrent lower respiratory tract infection or sirolimus-induced pneumonitis. No deaths and no pneumothoraces occurred during therapy.

**Conclusions:**

Our data suggest that sirolimus might be considered as a therapeutic option in rapidly declining LAM patients. However, sirolimus administration may be associated with severe respiratory adverse events requiring treatment cessation in some patients. Moreover, discontinuation of sirolimus is mandatory prior to lung transplantation.

## Background

Lymphangioleiomyomatosis (LAM) is a rare lung disease which almost exclusively affects young women. LAM occurs in an isolated form as sporadic LAM or in association with the genetic disease tuberous sclerosis complex (TSC). The pulmonary manifestation of LAM is characterized by infiltration of smooth-muscle cells and formation of parenchymal cysts. It results in dyspnea on exertion due to airflow obstruction, recurrent pneumothoraces and less frequently chylous pleural fluid collections. Approximately 40% of patients with sporadic LAM and more than 80% of TSC patients develop angiomyolipoma mainly of the kidneys with a risk of hemorrhage and renal failure. Genetic analyses suggest that cells of pulmonary LAM lesions and renal angiomyolipoma derive from a common source [1,2].

Treatment options include supportive use of bronchodilators, oxygen supplementation and specific surgical or interventional procedures for pneumo- and chylothorax or renal lesions, respectively. Moreover, avoidance or reduction of oestrogen exposure and administration of progesterone analogues have been used without clear evidence of therapeutic benefit. Nevertheless, since progressive pulmonary LAM or therapy refractory pneumothoraces ultimately lead to respiratory failure, lung transplantation remains the only available therapeutic option for end-stage LAM in carefully selected patients [3-5].

However, the identification of abnormal signalling in the TSC 1/2 genes resulting in constitutive activation of the mammalian target of rapamycin (mTOR) pathway has inspired studies to investigate the effect of mTOR inhibition with sirolimus in this disease [6]. The focus of these trials was primarily to demonstrate the ability of sirolimus to reduce renal angiomyolipoma volume [7,8]. However, the prospective open-label study of Bissler et al. provided some evidence in eleven patients suggesting that suppression of mTOR signalling might as well constitute a beneficial treatment option for pulmonary involvement in LAM [7]. In contrast, interim findings in a multicenter trial presented by Davies et al. did not reveal improvement of pulmonary function in three of four patients with available data [8].

Inspired by these findings, we started sirolimus therapy based on an individual risk benefit assessment in patients with documented progressive pulmonary LAM referred to our center for lung transplantation evaluation. The aim of the present study is to report our experience with sirolimus in this cohort of deteriorating patients suffering from respiratory failure in the absence of established medical alternatives.

## Methods

### Patient population

From November 2006 through December 2009, 10 consecutive patients with progressive pulmonary LAM referred to our center for lung transplantation evaluation, were included in this study. A confirmed diagnosis of the LAM associated with TSC or sporadic LAM, the use of contraception, the absence of relevant pleural effusion and the presence of a minimum of three pulmonary function tests (PFT) with at least two tests performed in our center prior to initiation of sirolimus were required for inclusion [9-11]. Informed written consent was obtained from each subject. The study was performed in accordance with recommendations of the local board on medical ethics at Ludwig Maximilians University of Munich.

### Sirolimus therapy and follow-up

Sirolimus was administered orally with a target trough level of 5 - 10 ng/ml. At the beginning sirolimus levels were measured twice a week and every other week thereafter after achievement of target level. Baseline was defined as start of sirolimus therapy and follow-up visits were scheduled every three months. At the time of initiation of sirolimus, hormone therapy including progesterone was discontinued.

Pulmonary function tests (PFT) including spirometry, body plethysmography, single breath diffusing capacity for carbon monoxide (DL_CO_) and blood gas analysis in arterialized capillary blood from the ear lobe while breathing room air were performed at baseline and during follow-up [12]. Parameters were calculated as percent of predicted [13]. Assessment of reversible airflow obstruction was conducted prior to baseline. In case of positive response, bronchodilator therapy was initiated before baseline measurement and continuously administered throughout the study period. The distance covered in 6 minutes (6-MWD) was measured according to the American Thoracic Society statement at baseline and after six months of sirolimus therapy [14]. Thoracic imaging was only performed in case of new respiratory symptoms, decline from baseline forced expiratory volume in 1 second (FEV_1_) or progressive hypoxemia. Renal angiomyolipoma size was not systematically followed. Moreover, routine follow-up included electrocardiogram, laboratory testing for red and white cell count, creatinine, electrolytes, and liver enzymes.

### Statistical Analysis and assessment of response to sirolimus therapy

Data analysis was performed retrospectively without pre-specified endpoints based on functional outcome or lung function testing. Statistics were calculated using SPSS Statistics software version 17.0. for Windows (SPSS Inc., Chicago, IL). Given the variable natural course of the disease assessing response to therapy is difficult in individual patients. Therefore, we plotted all available values of FEV_1 _measured before and after the therapeutic intervention over time and obtained the related slopes by linear regression analysis expressed as mean rate of change of FEV_1 _in ml/day. Benefit of sirolimus therapy was defined by comparison of the slopes before and after the intervention. The pre- and post-treatment slopes and outcomes were compared using a two-tailed paired Student's t-tests. Data are presented as mean ± SEM (standard error of the mean) or as individual values. Results were considered statistically significant at p < 0.05.

## Results

### Patient Cohort

A total of ten female patients were included in this study (table 1). Eight patients were diagnosed with sporadic LAM only and two patients had LAM in association with TSC (patient #2, #7). Mean age at the time of enrolment was 42.4 ± 11.9 years with an average time of 4.6 ± 2.9 years (range 0.8 - 10.4 years) since establishment of diagnosis. Progressive dyspnea on exertion was the primary event leading to the diagnosis in seven cases and recurrent spontaneous pneumothoraces in three patients. Prior to enrollment, 50% of the patients have been treated with bronchodilators and 30% received progesterone derivates. No new bronchodilator therapy was instituted at the time of enrolment.

**Table 1:** Characteristics of 10 patients with lymphangioleiomyomatosis

Pat. ID	age* (years)	sirolimus therapy (days)	presence of angiomyolipoma	No. of pregnancies	Smoking history	No. of pneumothoraces
1	47	218	no	0	no	0

2	39	185	yes	1	no	1

3	28	325	no	0	no	2

4	33	758	no	1	no	1

5	49	749	no	3	no	1

6	39	817	yes	0	no	2

7	48	266	yes	0	yes	0

8	29	191	no	2	yes	3

9	51	295	no	0	no	0

10	58	854	yes	0	yes	0

*at time of enrollment

### Impact of sirolimus on pulmonary function and functional outcome

Time of observation with available pulmonary function data before initiation of sirolimus therapy was 21.3 ± 5.2 months (range 6.0 - 47.7) and follow-up time after start of sirolimus was 12.1 ± 2.81 months (range 6.1 - 28.1). The mean number of available lung function tests before sirolimus therapy was 6.0 ± 2.4/patient (range 3 - 15) and 5.9 ± 1.2/patient (3 - 11) during therapy.

**Figure 1:**
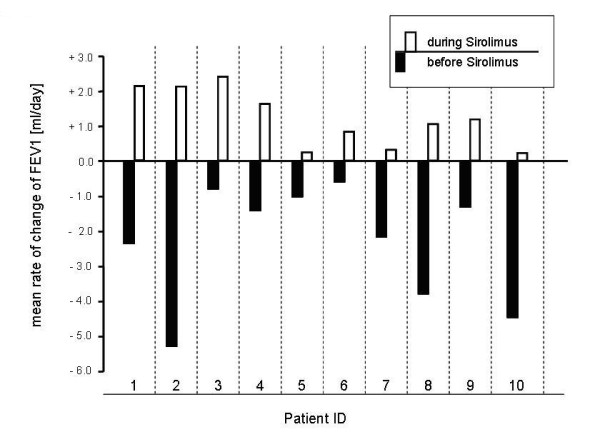
**Mean rate of change of FEV**_**1 **_**before and during sirolimus therapy**. Bars show the individual comparison of mean rate of change (ml/day) of FEV_1 _(forced expiratory volume in 1 second) before (black) and during (white) sirolimus therapy for ten patients.

**Figure 2:**
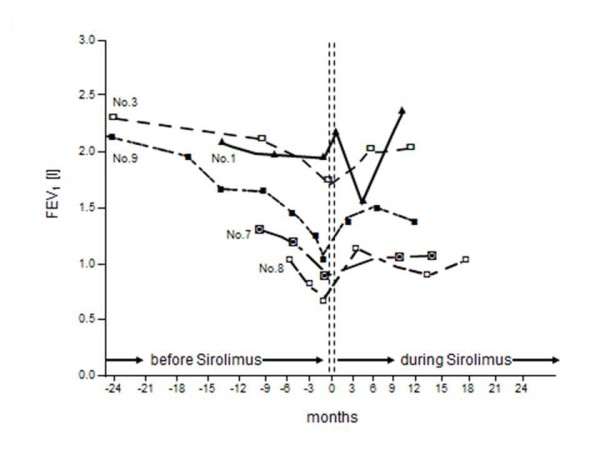
**Changes of FEV**_**1 **_**before and during sirolimus therapy**. Serial individual values of FEV_1 _(forced expiratory volume in 1 second) before and during sirolimus therapy in ten patients.

Figure 1 depicts the individual mean rate of change of FEV_1 _before and after initiation of therapy and figure 2, and 3 demonstrates the individual course of FEV_1 _with and without sirolimus. The overall mean loss of FEV_1 _amounted to -2.30 ± 0.52 ml/day before therapy. During the treatment with sirolimus, patients demonstrated a significant mean FEV_1 _gain of 1.19 ± 0.26 ml/day (p = 0.001). However, further analysis revealed no significant positive or negative correlation between the rate of change of FEV_1 _before and during sirolimus therapy or baseline FEV_1 _and relative increase of FEV_1 _after initiation of sirolimus, respectively (p > 0.05 each).

**Figure 3:**
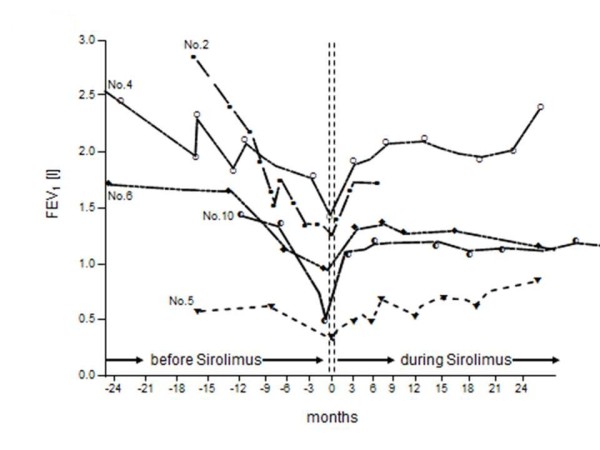
**Changes of FEV**_**1 **_**before and during sirolimus therapy**. Serial individual values of FEV_1 _(forced expiratory volume in 1 second) before and during sirolimus therapy in ten patients.

Mean FEV_1 _and Forced Vital Capacity (FVC) at baseline were 1.12 ± 0.15 l (36.1 ± 4.5%pred.) and 2.47 ± 0.25 l (69.2 ± 6.5%pred.), respectively. Follow-up pulmonary function tests revealed a significant increase of FEV_1 _and FVC at three and six months during sirolimus therapy in comparison to baseline values (3 months ΔFEV_1_: 220 ± 82 ml, p = 0.024; 6 months ΔFEV_1_: 345 ± 58 ml, p = 0.001); (3 months ΔFVC: 360 ± 141 ml, p = 0.031; 6 months ΔFVC: 488 ± 138 ml, p = 0.006). However, we detected no significant difference between three and six months measurements (table 2). Moreover, no significant changes of mean total lung capacity (TLC) after three months (6.57 ± 0.32 l, 125 ± 7.1%pred., p = 0.48) and 6 months (6.64 ± 0.38 l, 126.8 ± 8.7%pred., p = 0.67) in comparison to baseline values (6.11 ± 0.27 l, 116 ± 5.3%pred.) were noted.

**Table 2:** Pulmonary function and 6-Minute-walk-distance characteristics of 10 patients with lymphangioleiomyomatosis

Pat. ID	**FEV**_**1 **_**(l/% pred.)**								FVC (l/% pred.)							
	**Baseline**		**3 months**		**p**	**6 months**		**p**	**baseline**		**3 months**		**p**	**6 months**		**p**

1	1.79	62.0	1.39	49.0		2.22	77.6		2.65	77.4	2.75	81.4		3.17	93.2	
							
2	1.51	44.8	1.89	56.1		1.88	55.3		3.02	78.3	3.27	84.8		3.26	84.1	
							
3	1.69	44.3	1.86	48.7		1.98	51.9		2.90	66.1	2.99	67.3		3.12	71.4	
							
4	1.39	43.7	1.84	58.4		2.03	64.4		2.58	71.7	3.06	85.5		3.63	99.3	
							
5	0.34	12.3	0.53	19.1		0.64	23.3		0.78	24.9	1.58	49.6		1.54	48.1	
							
6	1.00	29.8	1.35	41.1		1.41	42.9		2.86	74.1	2.91	76.3		3.56	93.3	
							
7	0.93	34.4	1.22	45.1		1.11	41.3		3.17	99.0	2.99	94.2		2.99	94.2	
							
8	0.85	25.8	1.03	31.3		1.05	31.9		2.38	63.4	3.35	89.1		2.54	67.2	
							
9	0.95	27.6	1.22	41.6		1.30	44.4		2.98	88.5	2.94	86.4		3.18	94.2	
							
10	0.49	22.4	1.03	47.0		1.08	48.9		1.40	52.3	2.48	90.6		2.61	95.5	

**Mean ± SEM**	**1.12 ± 0.15**	**36.1 ± 4.5**	**1.34 ± 0.14**	**44.0 ± 3.7**	**0.024**	**1.47 ± 0.17**	**48.1 ± 4.9**	**0.001**	**2.47 ± 0.25**	**69.2 ± 6.5**	**2.83 ± 0.16**	**80.2 ± 4.2**	**0.031**	**2.96 ± 0.19**	**83.9 ± 5.25**	**0.006**

Baseline readings revealed a severe impairment of diffusing capacity (DL_CO _28.0 ± 3.4%ped., table 3). However, there was a small but significant increase in DL_CO _after six months as compared to baseline measurements (ΔDL_CO_: 4.7 ± 1.2%pred., p = 0.004), whereas blood gas analysis did not demonstrate a significant change after three and six months (table 3). Five patients (#4, #5, #7, #9 and #10) were on long-term oxygen supplementation at the beginning of the study. In all but one patient (#4), oxygen therapy was continued throughout the study period. No additional patients required long-term oxygen therapy during the conduct of the study.

**Table 3:** Pulmonary function and 6-Minute-walk-distance characteristics of 10 patients with lymphangioleiomyomatosis

Pat. #	DLCO (% pred.)					**pO**_**2 **_**(mmHg)**					6 MWD (m)		
	**baseline**	**3 months**	**p**	**6 months**	**p**	**baseline**	**3 months**	**p**	**6 months**	**p**	**baseline**	**6 months**	**p**

1	46			51		68	67		68		700	730	
									
2	38	37		38		61	67		69		500	530	
									
3	37			40		62	67		69				
									
4	26	33		39		55	56		62		420	510	
									
5	15	12		18		52	41		48				
									
6	23	25		24		69	67		70		510	535	
									
7	12	15		19		55	50		56		300	370	
									
8	22			30		59			63				
									
9	34	36		36		60	62		56		525		
									
10	27	32		32		57	64		68			540	

**Mean ± SEM**	28.0 ± 3.4	27.1 ± 3.8	0.15	32.7 ± 3.3	0.004	59.8 ± 1.7	60.1 ± 3.1	0.91	62.9 ± 2.3	0.88	486 ± 65	535 ± 57	0.02

Definition of abbreviations: FEV_1 _= forced expiratory volume in 1 second, FVC = forced vital capacity, DL_CO _= single breath diffusing capacity for carbon monoxide, pO_2 _= partial pressure of oxygen, 6-MWD = 6-Minute-Walk-Distance, SEM = standard error of the mean.

Complete 6-MWD testing at baseline and after six months of therapy was only available in 5 patients. Only patients on long-term oxygen supplementation used oxygen during 6-MWD testing (patient #4 and #7). Subgroup analysis for these patients revealed a modest though significant increase in 6-minute walk distance at six months (Δ 6-MWD: 49.0 ± 13.1 m, p = 0.02, table 3).

### Adverse events

Aphthous ulcers, peripheral edema or deterioration of kidney function or hemorrhage were not reported. No patient experienced pneumothoraces or relevant pleural effusion during the administration of sirolimus and no deaths occurred at available follow-up.

However, lower respiratory tract infection was recorded in 5 patients while receiving sirolimus. There was no evidence for pneumonia in two of these patients. In these two cases, the clinically mild event occurred within four weeks after initiation of sirolimus. No antibiotic therapy, discontinuation of sirolimus or hospitalization was deemed necessary and the symptoms resolved without sequelae. Nevertheless, sirolimus therapy was permanently stopped because of recurrent signs of lower respiratory infections with fever and leucocytopenia requiring hospitalization and antibiotic therapy in three patients (no. 1, no. 7, no. 8) after 218, 266 and 191 days, respectively. A definite distinction between pneumonia and sirolimus associated pneumonitis was not established and a pathogenic agent was not identified. After discontinuation of sirolimus and empiric broad spectrum antibiotic therapy, white blood count and respiratory symptoms resolved without sequelae.

However, two of these three patients underwent successful transplantation because of progressive deterioration of lung function (patient no. 7) and therapy refractory bilateral pneumothoraces (patient no. 8) 58 days and 322 days after cessation of sirolimus, respectively.

## Discussion

Encouraged by reports revealing positive effects in some cases with pulmonary LAM, we decided to start sirolimus therapy in patients with impaired pulmonary function and progressive respiratory disease on an individual basis. Our data support and extend the previous observations suggesting that even in an advanced stage of the disease sirolimus can potentially improve lung function. In addition, we found improvement of submaximal exercise capacity assessed by 6-MWD in some patients. However, due to the fact that a significant number of data is missing and the 6-minute walk test is prone to error without confirmative measurements our findings do not allow to draw firm conclusions regarding a relevant functional benefit of sirolimus over time at this point.

In the absence of an effective alternative treatment, lung transplantation is an accepted therapy for end-stage pulmonary LAM and outcome data are comparable to those achieved for other indications. However, due to overall limited long-term survival, lung transplantation may not be considered as a cure for the rather young cohort of patients affected by LAM [15,16].

Given the fact that TSC1 and TSC2 proteins regulate signalling through the mTOR pathway and the antiproliferative effects of sirolimus on smooth muscle cell growth, mTOR inhibition has emerged as a promising target for therapeutic interventions in pulmonary LAM [17].

This notion is supported by the results of Bissler et al., demonstrating a mean increase of FEV_1 _from baseline of approximately 120 ml after six and twelve months of sirolimus therapy in eleven LAM patients. However, only seven patients had abnormal lung function at the time of enrolment (moderate airflow obstruction in three patients and severe reduction of FEV_1 _in four patients) in this very important trial and no rate of decline prior to initiation of therapy was reported [7]. The present report confirms and extends these findings, in that we found significant improvement of lung function in a subset of patient with severe airflow obstruction and documented functional deterioration. Our findings are in line with a case report of Taille and co-workers, demonstrating a gain of 570 ml in FEV_1 _within six months of sirolimus therapy for pulmonary LAM starting from a FEV_1 _baseline of 32% predicted [18].

In contrast, interim data from an ongoing trial of sirolimus in Great Britain did not indicate an improvement of pulmonary function in four LAM patients with mild to severe airflow obstruction despite sirolimus therapy for twelve months [8]. In this respect, the accompanying editorial of Paul and Thiele in the New England Journal of Medicine provides a valuable insight into the molecular rationale for sirolimus therapy [19]. Nevertheless, the authors point out that the clinical effects of a pharmacological treatment for somatic mutations of TSC1-TSC2 complexes have to be very variable by nature. However, given the small sample size in our own work and the studies of Bissler et al. and Davies and colleagues, even differences in statistical techniques to assess response to therapy may be a crucial factor contributing to outcome discrepancies [7,8]. Another possible explanation for the lack of a significant effect of sirolimus may be the fact that in the study of Davies et al., adverse events resulted in all but one patient in periods of dose reduction or cessation [8]. We therefore speculate that in this trial mean exposure to the study drug was not enough to achieve an effect on pulmonary LAM. Unfortunately, significant adverse events including hospitalization due to relevant lower respiratory tract infections or sirolimus-induced pneumonitis have also been frequently observed in our patients and required termination of sirolimus even in the presence of initial functional improvement during therapy. Moreover, the occurrence of pneumothoraces as a potential late complication of sirolimus therapy warrants careful monitoring in future studies. Of note, we speculate that due to the lack of a rigorous assessment, our study might have missed a substantial number of minor adverse events and therefore underestimates the overall negative impact of sirolimus administration. Beyond that, it must kept in mind that discontinuation of the drug within several weeks prior to lung transplantation is mandatory in order to avoid dehiscence of the bronchial anastomosis due to impaired wound healing.

In addition the rate of progression of disease is variable with some patients experiencing a long term course lasting for decades and partially reversible airflow obstruction further complicating outcome assessment. In a large cohort of LAM patients with initially only mild impairment of pulmonary function (mean FEV_1 _75.2%pred.), the average rate of change in FEV_1 _was reported to be only -75 ml/year [20,21]. In contrast, our own small study population demonstrated a projected overall loss of 840 ml per year with an initial FEV_1 _of 36.0%pred. and severe impairment of diffusing capacity. According to the study of Taveira-DaSilva and colleagues, the most important predictors for further functional decline are initially low FEV_1 _and severely reduced DL_CO_. Therefore, it is unlikely that the observed positive functional responses for both FEV_1 _and FVC readings in our patients are explained by individual variable course of the disease or reversal of airflow obstruction alone [22].

Moreover, our therapeutic approach is supported by the results of the recently published MILES-trial [23]. McCormack and colleagues demonstrated stabilization and to some extent improvement of lung function parameters in LAM patients with moderate lung impairment. Of note, sirolimus was associated with an acceptable safety profile over a treatment period of twelve months in comparison to placebo. Nevertheless, due to the lack of a predefined loss of FEV_1 _as inclusion criteria, the subset of patients benefiting most from this medical intervention remains to be established.

However, we have to take into consideration that the use of historic pulmonary function tests in our study increased the risk for a lack of standardisation especially for bronchodilator testing. In addition, we acknowledge that the main reason for referral of LAM patients to our center was evaluation for lung transplantation. So, these limitations might have resulted in a significant selection bias overestimating the rate of actual FEV_1 _loss and the impact of sirolimus treatment in comparison to the overall LAM population. Moreover, despite the fact that progesterone seems not to be effective in reducing the decline of lung function in LAM patients, we cannot rule out the possibility that withdrawal of progesterone therapy might have influenced the subsequent course in some patients [21].

## Conclusions

We clearly recognize the inherent limitations of our report with respect to our non-prospective design, lack of a control group, limited sample size and short follow-up time. Despite the substantial risk of hemorrhage and renal failure in case of angiomyolipoma, these complications can usually be managed with medication, dialysis or renal transplantation with an acceptable long-term outcome. In contrast, the prognosis for patients with pulmonary LAM can be very limited in case of disease progression given the overall limited survival benefit achieved by lung transplantation. The present study demonstrates that sirolimus administration for pulmonary LAM can be associated with serious adverse events. Nevertheless, our data suggest that the use of mTOR inhibitors might be considered as a potential therapeutic option in carefully monitored, rapidly declining LAM patients. However, this report first of all highlights the urgent need for further research addressing the efficacy, safety, and dosing of sirolimus in this population [24].

In this respect, future trials are still necessary to guide management of pulmonary LAM and prevent the routine use of potentially harmful therapies.

## Abbreviation list

DL_CO_: single breath diffusing capacity for carbon monoxide; FVC: Forced Vital Capacity; FEV_1_: forced expiratory volume in 1 second; LAM: Lymphangioleiomyomatosis; mTOR: mammalian target of rapamycin; 6-MWD: distance covered in 6 minutes; PFT: Pulmonary function tests; SEM: standard error of the mean; TSC: tuberous sclerosis complex.

## Competing interests

The authors declare that they have no competing interests.

## Authors' contributions

CN and ALH designed the study, analyzed and interpreted the data and wrote the manuscript. PH, VAH, IF, SL, WvW, TM, CB, HL, RB, and GZ collected and analyzed data. JB designed the study, analyzed and interpreted the data. All authors read and approved the final manuscript.
